# Identification of differentially expressed mRNAs and miRNAs in spermatozoa of bulls of varying fertility

**DOI:** 10.3389/fvets.2022.993561

**Published:** 2022-10-05

**Authors:** Eimear M. Donnellan, Jean-Philippe Perrier, Kate Keogh, Miriam Štiavnická, Caitríona M. Collins, Elaine M. Dunleavy, Eli Sellem, Naomi C. Bernecic, Patrick Lonergan, David A. Kenny, Sean Fair

**Affiliations:** ^1^Laboratory of Animal Reproduction, Department of Biological Sciences, Biomaterials Research Cluster, Faculty of Science and Engineering, School of Natural Sciences, Bernal Institute, University of Limerick, Limerick, Ireland; ^2^Animal and Bioscience Research Department, Animal and Grassland Research and Innovation Centre, Teagasc, Ireland; ^3^Technical University of the Shannon, Athlone, Co. Westmeath, Ireland; ^4^Centre for Chromosome Biology, Biomedical Sciences, National University of Ireland, Galway, Ireland; ^5^ALLICE, Innovation and Development, Paris, France; ^6^School of Agriculture and Food Science, University College Dublin, Dublin, Ireland

**Keywords:** bovine, spermatozoa, RNA, male fertility, miRNA

## Abstract

Bulls used in artificial insemination, with apparently normal semen quality, can vary significantly in their field fertility. This study aimed to characterize the transcriptome of spermatozoa from high (HF) and low (LF) fertility bulls at the mRNA and miRNA level in order to identify potential novel markers of fertility. Holstein-Friesian bulls were assigned to either the HF or LF group (*n* = 10 per group) based on an adjusted national fertility index from a minimum of 500 inseminations. Total RNA was extracted from a pool of frozen-thawed spermatozoa from three different ejaculates per bull, following which mRNA-seq and miRNA-seq were performed. Six mRNAs and 13 miRNAs were found differentially expressed (*P* < 0.05, FC > 1.5) between HF and LF bulls. Of particular interest, the gene pathways targeted by the 13 differentially expressed miRNAs were related to embryonic development and gene expression regulation. Previous studies reported that disruptions to protamine 1 mRNA (*PRM1*) had deleterious consequences for sperm chromatin structure and fertilizing ability. Notably, *PRM1* exhibited a higher expression in spermatozoa from LF than HF bulls. In contrast, Western Blot analysis revealed a decrease in PRM1 protein abundance for spermatozoa from LF bulls; this was not associated with increased protamine deficiency (measured by the degree of chromatin compaction) or DNA fragmentation, as assessed by flow cytometry analyses. However, protamine deficiency was positively and moderately correlated with the percentage of spermatozoa with DNA fragmentation, irrespective of fertility group. This study has identified potential biomarkers that could be used for improving semen quality assessments of bull fertility.

## Introduction

The advent of genomic selection (1), has enabled more reliable identification of genetically elite bulls for use in artificial insemination (AI) programs, as soon as they reach puberty. However, one of the associated problems is that it provides little time for test inseminations prior to widespread use of the bulls' semen. While AI centers perform robust laboratory based semen quality controls, primarily based on motility and morphological based parameters of spermatozoa post-thawing, bulls can still vary significantly in their field fertility. Indeed, there can be up to 25% variation in conception rates among bulls used commercially for AI, all of which have passed routine semen quality controls (2). This has led to interest in the use of more detailed assessments of sperm kinematics using computer-assisted sperm analysis (CASA) as well as cellular function using flow cytometry. However, despite various multivariate and statistical approaches being developed, no single test nor combination of tests can reliably and consistently predict field fertility. This is evident from the work of both Sellem, et al. (3) and Bernecic, et al. (4) which could explain approximately 40 and 47%, respectively, of the variation in Holstein bull fertility through the assessment of flow cytometry and CASA-based parameters. A recent study by Narud, et al. (5) increased the explained variation in bull field fertility to 59% by adding the measurement of intracellular metabolites and selected trace elements (such as amino acids, Fe and Zn) in the spermatozoa of Norwegian Red Bulls (5). This suggests that other factors, such as specific biochemical and/or molecular characteristics of spermatozoa could explain some of the remaining variation, and their assessments could improve bull fertility prediction.

The acquisition of the full fertility potential of males post puberty requires a complex reorganization of the genomic and epigenomic architectures of sperm cell precursors during spermatogenesis and spermiogenesis, which involves the sequential transcription of thousands of genes. Recent advances in transcriptomics have revealed that mature spermatozoa not only carry the paternal haploid genome, but also deliver various types of RNAs into the oocyte, including messenger RNAs (mRNAs), as well as long and small non-coding RNAs, such as transfer RNAs (tRNAs), ribosomal RNAs (rRNAs), Piwi-interacting RNAs (piRNAs), and microRNAs (miRNAs) (6, 7).

While once considered as only remnant transcripts produced during spermatogenesis, evidence now suggests that mRNAs are involved in capacitation, fertilization, and early embryogenesis (8). Numerous studies have been conducted to decipher the role of mRNAs in bull fertility, and have reported associations with the expression of certain mRNAs to the quality of semen and sire conception rate, such as the transcripts from genes *CRISP2, CCT8* and *PEBP1* (9), *AK1, IB5, TIMP2* and *PLCz1* (10) *PRM1* (11), *ADIPOQ, AR1* and *AR2* (12) *BMP2* and *TRADD* (13), *AQP7* (14), or *CB1* and *FAAH* (15). In a recent study using a RNA-seq approach, Card and colleagues identified 3,227 and 5,366 transcripts differentially expressed, respectively, in spermatozoa between high and low bull fertility populations (16).

While the transcription of mRNA is arrested by the time developing germs cells reach the late spermatid stage (17), accumulation of non-coding RNAs occurs both during spermatogenesis and during epididymal transit through the incorporation of epididymosomes (17), and it has been demonstrated that they play a fundamental role in regulating gene expression during early embryo development (18, 19). In particular, miRNAs function in RNA silencing and post-transcriptional regulation of gene expression. Similarly, to mRNAs, differential expression of miRNAs has been identified between bulls which differ in terms of fertility, such as bta-miR-502-5p and bta-miR-1249-3p (20), or bta-miR-15a and bta-miR-29b (21). Capra et al. (22) identified 83 differentially expressed miRNAs between bulls with high and low motile spermatozoa, with these miRNAs targeting biological pathways related to apoptosis. Recently, Turri, et al. (23) reported 13 differentially expressed miRNAs between HF and LF with miR-423-3p highly expressed among LF bulls, which is known to be associated with severe asthenozoospermia in human spermatozoa. In the same study, miR-191 had increased expression in HF bulls and has previously been positively associated with the fertilization rate of blastocysts (24).

While numerous studies have been conducted to catalog mRNAs and miRNAs which are involved in bull fertility, in many studies the fertility phenotype is unreliable. Factors contributing to this inaccuracy include (i) lack of sufficient divergence in the mean bull field fertility estimates employed, (ii) estimates based on too few inseminations, and (iii) in some cases RNA extracted from a single ejaculate, with well-established inter-ejaculate variation not considered (25). The population of bulls in this study was based on an adjusted sire fertility model calculated from at least 500 inseminations per bull with an average calving rate difference between high and low fertility groups of 13.1 % (26). To account for inter-ejaculate variation, a pool of straws from three ejaculates per bull were used for analysis. The aim of this study was to examine sperm mRNA and miRNA fingerprints and associated biological pathways between high and low fertility bulls.

## Materials and methods

### Ethical approval

All protocols were in accordance with the Cruelty to Animals Act (Ireland 1876, as amended by European Communities regulations 2002 and 2005) and the European Community Directive 86/609/EC. Ethical approval was not required as the semen was collected from animals under routine conditions as part of commercial practice and subset of semen straws were subsequently donated to this research project.

### Animals and semen collection

Mature Holstein-Friesian bulls with high (HF; *n* = 10) or low (LF; *n* = 10) fertility were selected from a population of 1,665 AI bulls. Bull fertility was based on adjusted fertility scores (26), calculated from a record of at least 500 inseminations (mea*n* = 13,292, mi*n* = 519, max = 100,288). HF bulls showed an average adjusted fertility score of +6.5%, whereas LF bulls showed an average fertility score of −6.6%. Sire fertility was defined as pregnancy to a given service identified retrospectively either from a calving event or where a repeat service (or a pregnancy scan) deemed the animal not to be pregnant. These raw data were then adjusted for factors including semen type (frozen, fresh), cow parity, month of service, day of the week when serviced, service number, cow genotype, herd, AI technician, and bull breed. The adjusted sire fertility index given for each bull was then weighted for the number of service records, resulting in an adjusted calving rate. The mean of the population was zero. Semen was collected at two AI centers in Ireland via an artificial vagina, frozen in 0.25 ml straws using a programmable freezer (Digitcool, IMV Technologies, L'Aigle, France) and stored in liquid nitrogen pending further analysis. All ejaculates passed quality control checks for motility (post thaw motility of >50% as assessed subjectively) and morphology (>70% normal sperm).

### Total RNA extraction

From each bull, two straws from three different ejaculates were pooled (i.e., 6 straws in total) to minimize transcript expression profile biases. The pools were centrifuged at 2,400 *g* for 7 min at room temperature (RT), and the supernatant discarded. The sperm pellet was resuspended in 1.5 ml somatic cell lysis buffer (SCLB, 0.1% SDS + 0.5% Triton X-100 in RNase-free H_2_O) to obtain a pure sperm cell population (27). After centrifugation and SCLB supernatant removal, the pellet was washed with phosphate-buffered saline (PBS). Total RNA was then extracted as described previously (7), with modifications. After washing, the pellet was resuspended in 98 μl RLT buffer (Qiagen, Hilden, Germany) with 2-mercaptoethanol. Then, 1 ml of TRIzol was added to the sperm pellet and homogenized by vortexing for 1 min, and incubated for 5 min at RT. To the lysate, 100 μl chloroform was added and mixed vigorously by hand for 15 s, and tubes were allowed to stand at RT for 3 min. The mixture was centrifuged at 12,000 *g* for 15 min at 4°C. After centrifugation, the upper aqueous layer containing RNA was transferred to a new 1.5 ml tube, to which 100 μl chloroform was added and the same process redone. 500 μl of isopropanol and 25 μl of glycogen was added to the aqueous solution and mixed gently. The mixture was kept overnight at −20°C. After centrifugation at 12,000 *g* for 15 min at 4°C, the supernatant was discarded, and 1.5 ml of 75% ethanol was added to the RNA pellet and centrifuged at 12,000 *g* for 15 min, 4°C. Ethanol was removed, and the RNA pellet was air-dried for 10 min to remove traces of ethanol. The pellet was dissolved in 44 μl of RNase-free H_2_O. Then, removal of traces of gDNA was performed by treatment with DNase (TURBO DNase; Ambion, Austin, TX, USA) for 30 min at 37°C. To remove the enzyme and its buffer, the extraction process was performed again. In the end, and after two washing steps with 75% ethanol, total RNA was eluted in 12 μl RNase-free H_2_O. RNA concentration was determined using the Qubit RNA HS Assay kit on the Qubit 4.0 Fluorometer (Invitrogen, Waltham, MA, USA).

### Quality controls before sequencing by RT-PCR

Studying sperm RNA primarily relies on extracting non-biased and good quality RNAs, which still presents several challenges (28, 29). As the quantity of RNA in spermatozoa is exceptionally low compared to somatic cells, on the femtogram scale, the quantity of both coding and non-coding RNAs in spermatozoa is about 200 fold lower than in somatic cells; Goodrich et al. (30) and the analysis of sperm RNAs is prone to bias. Potential sources of cellular contamination, such as epithelial cells, leucocytes, and immature diploid spermatocytes, need to be removed from the samples. Among the various methods available (31), we chose somatic cells lysis using SCLB treatment, since its efficacy is known (32). Sperm RNA purity was confirmed by RT-PCR, a method which we also employed to assess RNA quality after extraction, as spermatozoa do not contain intact 18S and 28S ribosomal RNA, preventing calculation of the RNA Integrity Number (RIN).

Three nanograms of total RNA was reverse-transcribed and amplified using the OneStep RT-PCR kit (Qiagen) according to the manufacturer's instructions and using 40 amplification cycles. Primers were designed using Primer-BLAST (33), with the bovine reference genome ARS-UCD1.2. Genomic DNA contamination was tested using intron-spanning primers specific to the bovine protamine 1 (*PRM1*) gene. The positive control was a purified bovine genomic DNA extracted from sperm cells, following a protocol previously described (34). Absence of leucocytes, epithelial and germ cell contamination was tested using primers targeting protein tyrosine phosphatase receptor type C (*PTPRC*), cadherin-1 (*CDH1*) and mast/stem cell growth factor receptor Kit (*KIT*), respectively, as these genes are not expressed in sperm cells. RNA extracted from bovine uterine samples was used as positive control. For each RT-PCR run, negative controls were added and consisted of RT-PCR mix without template RNA. All primers were supplied by Sigma (St. Louis, MO, USA), and their sequences are listed in Supplementary Table S2. The PCR products were separated by electrophoresis with 1.5% agarose, 0.5X Tris-acetate EDTA gel stained with SYBR safe gel stain (Invitrogen).

### mRNA and miRNA sequencing

Library preparations from total RNA and sequencing were carried out by GenomeScan (Leiden, The Netherlands). RNA concentration and the size distribution of the RNAs were determined using a Fragment Analyzer system (Agilent, Santa Clara, CA, USA). For mRNA-seq, library preparation was carried out using the NEBNext^®^ Single Cell/Low Input RNA Library Prep Kit for Illumina (NEB, Ipswich, MA, USA). For small RNA-seq, library preparation was performed using the NEBNext^®^ Multiplex Small RNA Library Prep kit for Illumina (NEB). Possible adapter dimers were removed using the Blue Pippin size selection. Both mRNA and small RNA libraries were sequenced on a NovaSeq6000 (Illumina, San Diego, CA, USA) according to manufacturer's instructions, using the following parameters: paired-end and 150 bp read length.

### mRNA-seq bioinformatics and data analysis

Raw sequencing reads were first checked for sequencing quality using FASTQC (version 0.11.8, https://www.bioinformatics.babraham.ac.uk/projects/fastqc/). Sequencing reads were then trimmed using Trimmomatic V0.30 (35) of Illumina TruSeq adapter sequences. Following removal of sequencing adapters, reads were aligned to the *Bos taurus* reference genome (ARS-UCD1.2 including the Y chromosome from Btau_5.1 assembly), using the read aligner TopHat [v2.0.14; (36)] with default settings. Based on the mapped locations in the read alignment files, the frequency of how often a read was mapped to a transcript was determined through HTSeq [v0.6.1.pl; (37)]. The number of read counts mapping to each annotated gene from HTSeq was then collated into a single file to be used for subsequent differential gene expression analysis. The R (version 2.14.1; (38)). Bioconductor package, EdgeR [version 3.26.7, (39)] was employed to undertake differential gene expression analysis of sequencing data. For this, mRNA reads were first converted to counts per million within EdgeR; any mRNA within samples that had less than one count per million (CPM) in at least half of the samples was subsequently removed from the analyses. For the retained mRNAs, their counts were normalized using the trimmed mean of M values (TMM) method. To test for differential mRNA expression between treatment groups, the normalized counts were modeled using a generalized linear model under a binomial distribution using moderated tagwise dispersions.

### miRNA-seq bioinformatics and data analysis

Raw sequence reads were firstly assessed for sequencing quality using FASTQC [version 0.11.8; (40)]. The Illumina sequencing adaptor was clipped off all the raw read sequences using Cutadapt [version 1.18, (41)]. Reads of lengths shorter than 15 bp, and longer than 28 bp were subsequently removed as short and long reads, respectively. The retained reads were then additionally filtered for other bovine short RNA species including ribosomal RNAs (rRNAs), transfer RNAs (tRNAs), small nuclear RNAs (snRNAs) and small nucleolar RNAs (snoRNAs) downloaded from https://rnacentral.org/. To profile miRNA expression in each sample, the miRDeep2 package [version 2.00.8, (42)] modules were used, together with the bovine reference genome (ARS1.2+y) and the known bovine mature miRNA sequences and their precursor sequences from the miRBase database [release 22.1, (43)]. The miRDeep2 mapper module (mapper.pl) was then used with default parameters to collapse reads of the sequences into clusters. Bowtie 1 [version 1.1.1, (44)] was then employed to align the collapsed reads to the indexed reference genome. Using default parameters, and input files including the reference genome, collapsed reads vs. reference genome alignment, known bovine mature miRNAs and their precursors sequences (including the hairpin structures), and *Bos taurus* (bta) as the species of interest, the miRDeep2 module (miRDeep2.pl) was used to quantify bovine miRNAs. Through this, the miRDeep2 quantifier module was used to quantify all expressed miRNAs in the sequence data, producing read counts for each individual sample.

Resultant read counts for each sample were merged into one file and subsequently assessed for differentially expressed miRNA using the R (v2.14.1) Bioconductor package, EdgeR (version 3.26.7) as previously described for mRNA. Target genes for differentially expressed miRNA were predicted using TargetScan [release 7.2, (45)], and the biological pathways in which the target genes are involved was revealed using the KEGG pathway analysis.

### Validation of the mRNA and miRNA-seq data by reverse-transcription—Quantitative PCR

To obtain sufficient RNA template for performing the validations of the mRNA-seq and miRNA-seq data by RT-qPCR, total RNA was extracted from a separate batch of straws, originating from the same ejaculates from the same bulls as for the mRNA-seq experiment.

RT-qPCR was carried out for five of the six differentially expressed genes (DEGs) that were identified by mRNA-seq: *PRM1, SCP2D1*, and *RBBP6*, and the 2 novel genes (no primers could be successfully designed for *SLC24A1*). RT reactions were performed using 20 ng of template RNA and the high-capacity cDNA reverse transcription Kit (Applied Biosystems, Waltham, MA, USA), following the manufacturers' instructions. Each sample reaction contained 1 μl of multiScribe reverse transcriptase, 2 μl of 10X RT random primers, 0.8 μl of 25X dNTP mix (100 mM), 2 μl of 10X RT buffer, 4.2 μl of nuclease free water and 10 μl of total RNA (at 2 ng/μl). The following program was used in a 2720 Thermal Cycler (Applied Biosystems): 10 min. at 25°C, 120 min. at 37°C, and finally 5 min. at 85°C. For real-time quantitative PCR (qPCR) reactions; primers were designed using Primer-BLAST (33) and with the bovine reference genome ARS-UCD1.2 (primers sequences can be found in Supplementary Table S3). Primer efficiencies were assessed using serial dilutions of pooled cDNA samples and were calculated to be >80% and <120 %. qPCR reactions were performed in triplicate using the TaqMan Fast Advanced Master Mix (Applied Biosystems) following the manufacturer's instructions. Each sample reaction contained 10 μl of 2X TaqMan Fast Advanced Master Mix, 1 μl of 10 μM primer mix, 7 μl of nuclease free water and 2 μl cDNAs. The following program was used: 20 s at 95°C, followed by 40 cycles of 3 s at 95°C and 30 s at 60°C in a 7500 Fast Real-Time PCR System v2.0.1 (Applied Biosystems). Resultant Ct values were then imported into GenEx software (v.5.2.7.44). Further processing of Ct values in GenEx included adjustment of Ct values to PCR primer efficiencies, averaging of qPCR repeats, normalization of expression values to those of the reference gene (*YWHAZ*) and calculation of relative gene expression values.

Due to low levels of expression, it was not possible to directly validate the differentially expressed miRNAs by RT-qPCR. Spermatozoa have low levels of miRNAs and the differentially expressed miRNAs were not the most abundant ones. Therefore, a technical validation of the miRNA-seq data was carried out by RT-qPCR on 2 miRNAs that were identified as highly expressed in all samples (bta-miR-100 and bta-miR-34c). bta-miR-125 was used as a reference miRNA as it was previously reported as being highly expressed in spermatozoa (46). This approach was similar to that taken by Sellem, et al. (7) and its aim was to demonstrate that the relative abundance of miRNAs is broadly consistent across miRNA seq and RT-qPCR. RT reactions were performed using 10 ng of template RNA and the TaqMan MicroRNA Reverse Transcription Kit (Applied Biosystems), following the manufacturer's instructions. Each sample reaction contained 1 μl of multiScribe reverse transcriptase, 3 μl of 5X stem-loop RT primer, 0.15 μl of 100 mM dNTPs, 1.5 μl of 10X RT buffer, 0.19 μl of RNase inhibitor (20 U/μl), 4.16 μl of nuclease free water and 5 μl of total RNA (at 2 ng/μl). The following PCR program was used in a 2720 Thermal Cycler (Applied Biosystems): 30 min at 16°C, 30 min at 42°C, and finally 5 min at 85°C. For qPCR reactions, primers were retrieved from TaqMan MicroRNA assay (Applied Biosystems). Primer efficiencies were assessed using serial dilutions of pooled cDNA samples and were calculated > 80 % and < 120 %. qPCR reactions were performed in triplicate using the TaqMan Fast Advanced Master Mix (Applied Biosystems) following manufacturers' instructions for TaqMan MicroRNAs Assays. Each sample reaction contained 10 μl of 2X TaqMan Fast Advanced Master Mix, 1 μl 20 X TaqMan MicroRNA Assay primers, 4.5 μl of nuclease free water and 4.5 μl cDNAs. The following qPCR program was used: 20 sec at 95°C, followed by 40 cycles of 3 s at 95°C and 30 s at 60°C; in a 7500 Fast Real-Time PCR System v2.0.1 (Applied Biosystems). For the analysis, bta-miRNA-125 was used to normalize the data. Statistical analysis was carried out using the Kruskal-Wallis test (R version 2.14).

### Spermatozoa nuclear protein acid extraction and western blotting

The most expressed sperm-specific gene in the RNAseq analysis was *PRM1*, which had higher expression in spermatozoa from LF than HF bulls. Therefore, the objective was to assess the protein PRM1 level in spermatozoa from the HF and LF bulls. Sperm nuclear proteins were extracted according to de Yebra and Oliva (47). For each bull, semen from three separate ejaculates was pooled and the sperm concentration was determined prior to processing. Semen samples were washed twice in cold PBS containing 6 mM EDTA and 1 mM PMSF, followed by hypotonic wash in ddH_2_O with 6 mM EDTA and 1 mM PMSF. Sperm nuclear proteins were denatured for 30 min at RT in PBS containing 6 mM EDTA, 1 mM PMSF, 2.4 M guanidine hydrochloride and 100 mM DTT. One ml of 100% ethanol (−20°C) was then added and the sample was centrifuged at 12,000 *g* for 10 min at 4°C. The supernatant was then removed and discarded, and the gel-like pellet was washed twice in 1 ml of 100% ethanol. The pellet was then resuspended in 0.5 M hydrochloric acid and the nuclear proteins were acid-solubilized under agitation for 15 min at 37 °C. The samples were centrifuged at 12,000 *g* for 10 min and the supernatant containing the solubilized nuclear proteins was retained. Trichloroacetic acid was added to a final concentration of 40% and precipitated nuclear proteins were collected by centrifugation at 20,000 *g* for 20 min at RT. The pellet was washed twice in 100% acetone containing 1% 2-mercaptoethanol and then air-dried. Pellets were snap frozen in liquid nitrogen and stored at −80°C. Purified nuclear proteins were resuspended in methylene blue sample buffer containing 6 M urea and 5% acetic acid and incubated for 1 h at RT prior to loading onto 16% poly-acrylamide gels containing 6 M urea and 5% acetic acid. For each bull, a volume corresponding to 110,000 sperm cells was analyzed. Gels were run under reverse polarity at 120–150 V for 100 min. Separated proteins were transferred to 0.2 μm pore size PVDF membrane in a 0.7% acetic acid transfer buffer for 25 min at 340 mA. Membranes were blocked in TBS-Tween (0.1%) containing 5% milk for 1 h at RT followed by overnight incubation at 4°C with anti-PRM1 antibody (mAb Hup-1N, BriarPatch Biosciences, $\frac{1}{2}$,000 dilution). The signal intensities of PRM1 protein bands from each bull were quantified using Bio-1D Analysis Software (Vilbur Lourmat Fusion Fx6 EDGE imaging system). Following background subtraction, the sum of pixel intensities in a fixed area surrounding each band was determined. Data were pooled from two biological replicates, each with three technical repeats.

### Flow cytometric assessment of protamine deficiency and DNA fragmentation

The assessment of protamine deficiency and DNA fragmentation were performed on a CytoFLEX flow cytometer from Beckman Coulter (Labplan; Dublin, Ireland). CytoFLEX daily quality control fluorospheres (Beckman Coulter) were used prior to each experiment to verify the optical alignment. A sperm-specific population was gated following identification with side and forward scatter. For all assessments, 10,000 events were recorded (unless otherwise stated) and the area of the signal pulse was used during data collation. The collection and preparation of data for analysis was performed using the CytExpert software. For those parameters measured, an ejaculate from a single bull (reference sample) was included for each assessment to monitor day-to-day variation. Chromomycin A3 (CMA3) and Acridine Orange (AO) were sourced from Sigma Aldrich and Invitrogen, respectively.

CMA3 labeling of G-C regions of DNA was employed as an indirect assessment of protamine deficiency (as measured by the degree of chromatin compaction) (48). For this assessment, a protocol adapted from Fortes et al. (49) was used. Briefly, frozen-thawed spermatozoa were washed twice with PBS free of Ca^2+^ and Mg^2+^ (PBS; pH 7.0) *via* centrifugation (500 x g, 5 min). As a positive control, a reference sample was incubated with 5 mM DTT for 15 min at 37°C prior to washing with PBS. Following washes, samples were resuspended to 50 x 10^6^ sperm/mL with 0.25 mg/mL CMA3 in McIlvaine's buffer (17 mM citric acid, 164 mM Na_2_HPO_4_, 10 mM MgCl_2_.6H_2_O; pH 7.0) and incubated at RT, in the dark for 1 h. Prior to flow cytometric assessment, spermatozoa were washed with PBS (500 x g, 5 min) to remove excess CMA3 and diluted to 5 x 10^6^ sperm/mL. CMA3 was excited by a 405 nm laser and detected with 525/40 nm band-pass filter. Three populations (low, medium and high CMA3 labeling) were detected with this fluorophore as observed in a previous study (49), with high CMA3 labeling being indicative of protamine deficiency. The susceptibility of sperm chromatin to DNA fragmentation was assessed using AO. As a positive control for DNA fragmentation, a reference sample was incubated with 0.8 M HCl for 5 min at 37řC prior to assessment. Using one straw from the same ejaculates as used for RNA extraction (20 bulls, 3 ejaculates per bull), samples were prepared and stained with AO according to the protocol described by Evenson and Jost (50). AO was excited using a 488 nm laser and green and red fluorescence was detected with a 525/40 nm or 690/50 nm band-pass filter, respectively. During data acquisition, the flow rate was adjusted to approximately 200 events/s and 5,000 events (in the sperm-specific gate) were recorded for analysis. The population with high red and low green fluorescence was identified as spermatozoa with high DNA fragmentation.

### Statistical analysis of the western blot and flow cytometry data

In all experiments, the individual bull was the experimental unit. Western blot data were normalized using MIN-MAX scaling and statistical analysis was carried out using the Student's unpaired *t*-test (PRISM 8 software). All statistical analyses for CMA3 and DNA fragmentation were performed using R. Fertility ranking was set as a fixed effect in the model, whereas bull and ejaculate were included as nested random effects. Normality and homoscedasticity of the residuals were assessed for all models by use of Shapiro-Wilk test and Bartlett's test, respectively. Identification of statistical outliers was assessed by Cook's Distance and observations were removed when necessary. Pairwise comparisons between high and low fertility were determined using a Tukey adjustment. To determine the relationship between CMA3 and DNA fragmentation, a Pearson's product-moment correlation was performed using all data from individual ejaculates collected from HF and LF bulls. With the exception of the correlation, all data are presented as the mean ± s.e.m.

## Results

### Sperm RNA yield, quality, and purity

**Figure 1:**
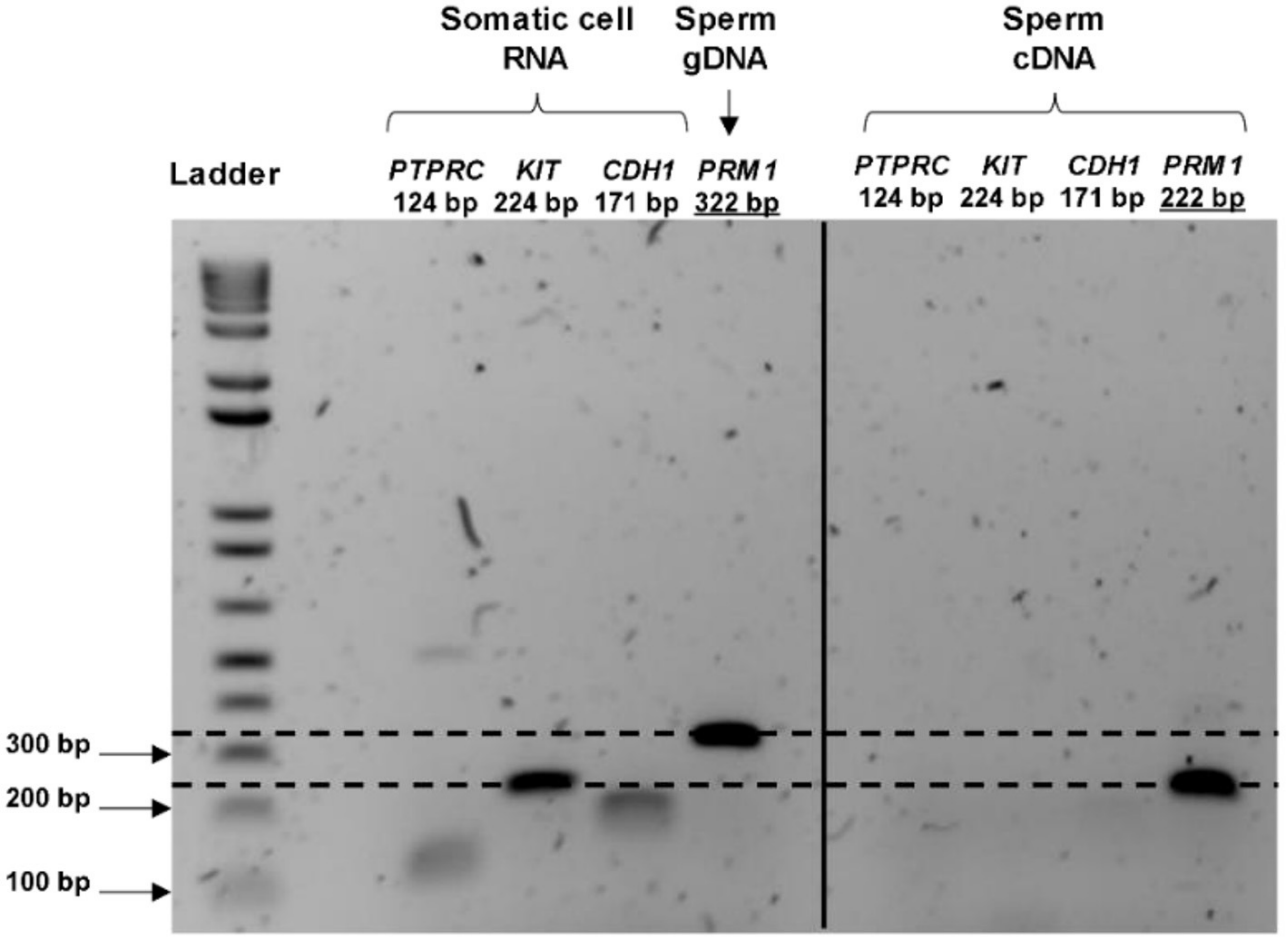
RNA purity control and validation of the presence of transcripts in bull spermatozoa. The cDNA from the control (cow uterine RNA) amplifies all the primers at the expected product size, whereas the cDNA synthesized from the sperm RNA amplified only for the sperm-specific primer (*PRM1*). *PRM1* primers were designed to span a 100 bp intron; therefore, the gDNA signal appeared at 322 bp for the control (gDNA extracted from spermatozoa), whereas mRNA appeared at 222 bp. Overall, these indicated the purity of the sperm RNA from other contaminating cells and sperm genome DNA.

Total RNA extraction from the pooled bovine straws resulted in a recovery of an average RNA concentration of 62.6 ± 42.7 ng per sample, from 83.0 x 10^6^ ± 32.4 sperm cells. Contamination with genomic DNA was not detected by RT-PCR in any of the tested samples, nor was any RNA contamination arising from leukocytes, germ cells or epithelial cells (as demonstrated by the lack of amplification of *PRM1* at 322 bp, *KIT, PTPRC*, and *CDH1* in spermatozoa, respectively; Figure 1). Electrophoretic profiles obtained before library preparation exhibited an absence of intact 18S and 28S rRNA peaks, and a peak fragment size at 115 bp (data not shown). Together, this demonstrated that only RNAs originating from sperm cells were present in the samples, and that there was an absence of gDNA contamination.

### Identification of the differentially expressed mRNAs between HF and LF bulls

Sequencing of the 20 libraries generated an average of 20.2 (±9.2) Gb of data, and an average of 67.4 million reads per sample (Table 1). On average, 91.9 (±0.7) % of the data showed a Q-score over 30 (i.e., a base-calling accuracy of 99.9%). There was no difference for any of the above parameters when comparing the HF and LF groups of bulls (*P* > 0.05). This eliminated the possibility of technical bias during RNA library preparation or sequencing that could affect subsequent results and indicated that the data were of overall good quality.

**Table 1:** Library characterization and mapping efficiency on the bovine genome (ARS-UCD1.2) of in mRNA-seq and miRNA-seq libraries.

**Group**		**High fertility**	**Low fertility**
	Yield (Gb)	21.3 ± 13.1	19.2 ± 1.8
mRNA-seq data	Number of read pairs analyzed (million)	71.1 ± 43.7	63.8 ± 6.0
	Number of bases with a Q-score >30 (%)	91.7 ± 0.7	92.1 ± 0.7
	Uniquely mapped reads (%)	67.0 ± 8.5	66.8 ± 11.3
	Ambiguous reads (%)	2.4 ± 0.3	2.3 ± 0.4
	Unmapped reads (%)	30.6 ± 8.7	30.9 ± 11.7
	Yield (Gb)	4.6 ± 1.3	4.3 ± 0.5
miRNA-seq data	Number of read pairs analyzed (million)	15.2 ± 4.3	14.4 ± 1.8
	Number of bases with a Q-score >30 (%)	81.7 ± 1.2	80.2 ± 2.0
	Reads <15bp (%)	21.2 ± 6.6	21.8 ± 7.3
	Mapped reads (%)	66.8 ± 8.4	66.4 ± 7.5
	Unmapped reads (%)	33.2 ± 8.4	33.6 ± 7.5

**Figure 2:**
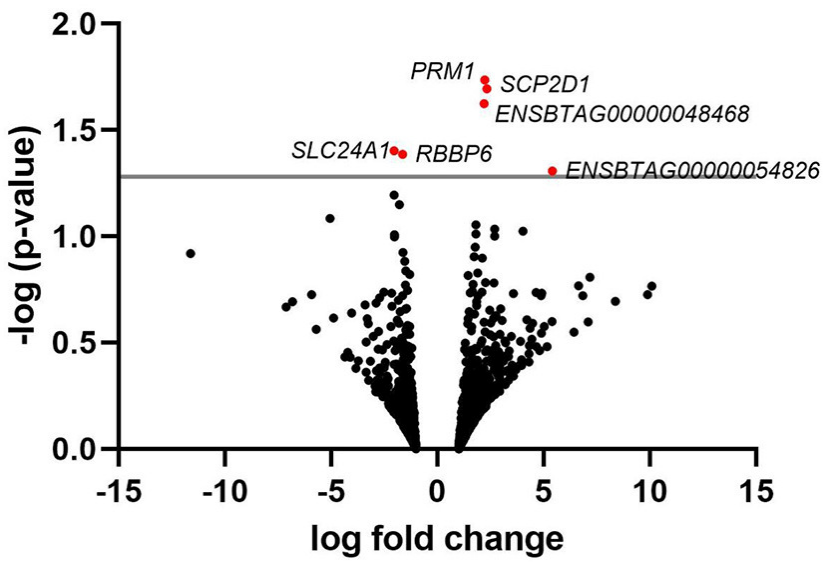
Volcano plot depicting an overview of differential mRNA expression between bulls of varying fertility. The red dots indicate genes deemed significantly differentially expressed between bulls of high and low fertility. Genes with a positive log fold change were upregulated in the low fertility group. The gray horizontal line depicts the *p*-value threshold cut-off (*P* < 0.05).

A list of the 20 most highly expressed mRNAs, unrelated to the fertility group, is given in Table 2, the most highly expressed being the sperm-specific gene *PRM1*. In total, we identified six differently expressed mRNAs between HF and LF, corresponding to the genes SCP2 sterol binding domain containing 1 (*SCP2D1*), protamine 1 (*PRM1*), solute carrier family 24 member 1 (*SLC24A1*), retinoblastoma binding protein 6 (*RBBP6*), and 2 novel genes, ENSBTAG00000048468 and ENSBTAG00000054826 (Figure 2). Genes *PRM1, SCP2D1*, and the two novel genes were found more highly expressed in the LF group, whereas *SLC24A1* and *RBBP6* were more highly expressed in the HF group.

**Table 2:** List of the 20 most highly expressed mRNAs.

**Gene symbol (Ensembl ID)**	**Gene name**	**Mean count**
*PRM1* (ENSBTAG00000021493)	Protamine 1	816,382
*GOLGA4* (ENSBTAG00000016563)	Golgin A4	554,717
- (ENSBTAG00000040318)	Novel gene	528,827
*BAZ2B* (ENSBTAG00000020654)	Bromodomain adjacent to zinc finger domain 2B	492,269
*EEA1* (ENSBTAG00000000421)	Early endosome antigen 1	488,231
*CHMP5* (ENSBTAG00000012383)	Charged multivesicular body protein 5	407,796
*CEP295* (ENSBTAG00000001902)	Centrosomal protein 295	360,010
*KIF5C* (ENSBTAG00000018125)	Kinesin family member 5C	315,901
*HMGB4* (ENSBTAG00000000335)	High mobility group box 4	311,026
*CCDC181* (ENSBTAG00000020264)	Coiled-coil domain containing 181	303,303
*CHD5* (ENSBTAG00000040477)	Chromodomain helicase DNA binding protein 5	222,768
*CHD2* (ENSBTAG00000051831)	Chromodomain helicase DNA binding protein 2	178,802
*SSRP1* (ENSBTAG00000000375)	Structure specific recognition protein 1	170,963
*RBBP6* (ENSBTAG00000009441)	RB binding protein 6, ubiquitin ligase	159,104
*TRA2B* (ENSBTAG00000001697)	Transformer 2 beta homolog	154,430
*CHD4* (ENSBTAG00000014734)	Chromodomain helicase DNA binding protein 4	154,060
*CCDC174* (ENSBTAG00000038298)	Coiled-coil domain containing 174	150,409
*BRD9* (ENSBTAG00000006971)	Bromodomain containing 9	142,487
*MYH10* (ENSBTAG00000021151)	Myosin heavy chain 10	135,613
*BAZ1A* (ENSBTAG00000020164)	Bromodomain adjacent to zinc finger domain 1A	131,847

### Identification of differentially expressed miRNAs

Sequencing of the 20 libraries generated an average of 4.4 (±1.0) Gb of data, and an average of 14.8 (±3.2) million reads per sample (Table 1). On average, 81.0 (±1.8) % of the data showed a Q-score over 30. As for the mRNA-seq data, there was no significant difference for any of the parameters when comparing the HF and LF bull, eliminating the possibility of technical bias.

**Figure 3:**
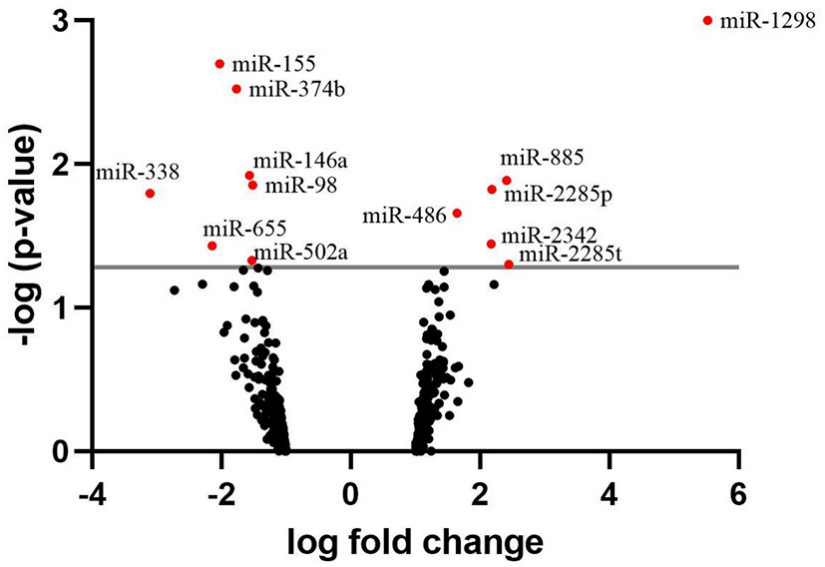
Volcano plot depicting an overview of differential miRNA expression between bulls of varying fertility. The red dots indicate genes deemed significantly differentially expressed between bulls of high and low fertility. Genes with a positive log fold change were upregulated in the low fertility group. The gray horizontal line depicts the *p*-value threshold cut-off (*P* < 0.05).

The identification of the differentially expressed miRNAs was carried out on 458 miRNAs that were found expressed across the samples. A list of the 20 most highly expressed miRNAs, unrelated to the fertility group, is given in Table 3. bta-miR-100 was the most highly expressed miRNA making up 39.3% of all miRNAs in sperm. In total, we identified 13 miRNAs where expression differed significantly between HF and LF (*p* < 0.05, FC>1.5), which are listed in Figure 3. Among them, six were found upregulated in the HF group, while the seven remaining were found downregulated in the HF group.

**Table 3:** List of the 20 most highly expressed miRNAs.

**miRNA name**	**Mean counts**	**% of miRNAs**
bta-miR-100	338,795	39.3
bta-miR-30d	36,838	5.9
bta-miR-21-5p	33,096	5.6
bta-miR-99a-5p	27,961	4.3
bta-miR-34c	32,641	3.6
bta-miR-191	19,618	3.1
bta-miR-27a-3p	17,983	2.8
bta-miR-2284x	15,428	2.3
bta-miR-186	15,159	2.1
bta-miR-148a	11,916	1.9
bta-miR-125b	16,198	1.7
bta-miR-22-3p	10,464	1.6
bta-miR-27b	10,235	1.6
bta-miR-3432a	8,904	1.4
bta-miR-375	7,175	1.1
bta-miR-128	7,422	1.1
bta-miR-16b	5,960	1.0
bta-miR-7	6,847	0.9
bta-miR-449a	7,931	0.8
bta-miR-204	7,619	0.8

### RT-qPCR validation of mRNA and miRNA RNA-seq

**Figure 4:**
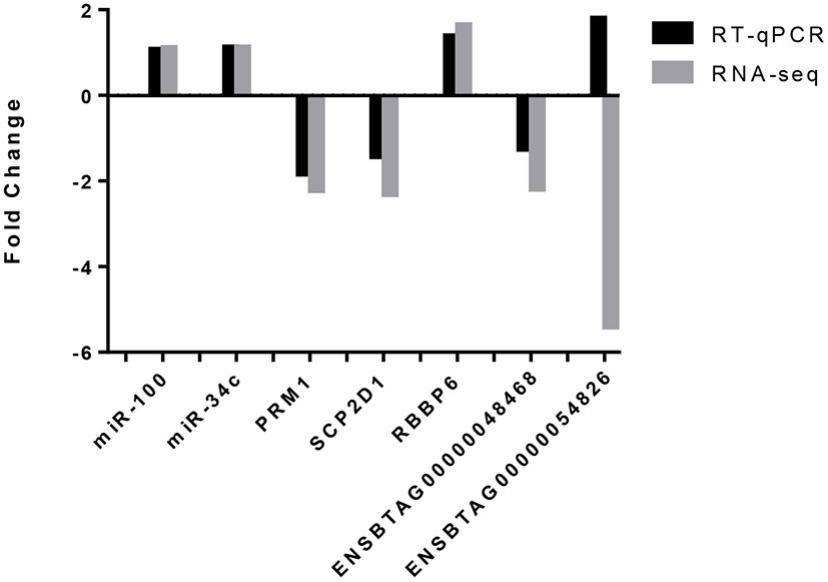
Validation of the mRNA-seq and miRNA-seq differentially expressed genes by RT-qPCR. Both miRNAs, miR-100 and miR-34c exhibited the same trend (an upregulation in the high fertility group) for RNA-seq and RT-qPCR. For four out of the five differentially expressed genes identified by mRNA-seq and assessed by RT-qPCR, the same trend was observed i.e., a downregulation of *PRM1, SCP2D1* and *ENSBTAG00000048468* as well as an upregulation for *RBBP6*. *ENSBTAG00000054826* did not observe the same trend.

Validation of the data was carried out by RT-qPCR on five of the six differentially expressed genes (DEGs): *PRM1, SCP2D1, RBBP6* and ENSBTAG00000048468 and ENSBTAG00000054826. Results are presented in Figure 4. For 4 out of 5 DEGs, RT-qPCR results showed the same trend than for the mRNA-seq results represented by a downregulation of *PRM1, SCP2D1* and *ENSBTAG00000048468* as well as an upregulation of *RBBP6* for the HF bulls. Concerning ENSBTAG00000054826, results were discordant with the mRNA-seq results.

To perform a technical validation of the miRNA-seq data, RT-qPCR was carried out on two miRNAs (bta-miR-100 and bta-miR-34c) which are highly expressed in the samples and are known to be highly expressed in bull spermatozoa (46). Results are also presented in Figure 4. Both miRNAs were expressed in all samples, which is in agreement with the miRNAseq data.

### Comparison between mRNA-seq and miRNA-seq results

As only six differentially expressed mRNAs were identified, pathway analysis could not be performed. For the differentially expressed miRNAs, an analysis of the transcripts they target was carried out using TargetScan. Three of them, bta-miR-655, bta-miR-2285p and bta-miR-2285t, have *RBBP6* as a common target, a DEG identified by mRNA-seq. A KEGG pathway analysis (51) was also carried out using the list of transcripts targeted by each differentially expressed miRNA, to identify if these targets participate in common biological pathways. It appeared that 5 of the differentially expressed miRNAs (bta-miR-2285p, bta-miR-98, bta-miR-155, bta-miR-374b, and bta-miR-486) target transcripts which are significantly involved in the same 3 biological pathways: signaling pathways regulating pluripotency of stem cells, mTOR signaling, and FoxO signaling (Table 4). For the mTOR and FoxO signaling pathways, two miRNAs are upregulated and two downregulated for HF bulls, but for the pathway regulating the pluripotency of stem cells, three out of four miRNAs are downregulated for HF bulls.

**Table 4:** Biological pathways targeted by the differentially expressed miRNAs between high fertility and low fertility bulls.

**Biological pathways**	**miRNA name**	Expression in high fertility bulls^*^	**Number of targets involved in the pathway**	**Corrected *p*-value (Benjamini)**
Signaling pathways regulating pluripotency of stem cells	bta-miR-2285p	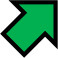	62	1.30E-07
	bta-miR-98	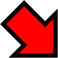	26	9.00E-07
	bta-miR-155	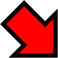	15	1.90E-04
	bta-miR-374b	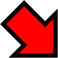	14	5.00E-02
mTOR signaling pathway	bta-miR-155	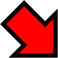	9	8.70E-04
	bta-miR-486	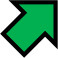	6	2.20E-03
	bta-miR-98	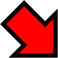	11	6.20E-03
	bta-miR-2285p	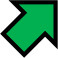	25	6.60E-03
FoxO signaling pathway	bta-miR-2285p	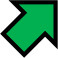	62	1.90E-08
	bta-miR-98	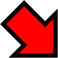	21	1.50E-04
	bta-miR-486	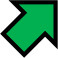	7	2.20E-03
	bta-miR-155	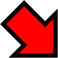	10	1.90E-02

* Direction of arrow indicates direction of expression in high fertility bulls.

### Comparison of PRM1 transcription and protein abundance levels

**Figure 5:**
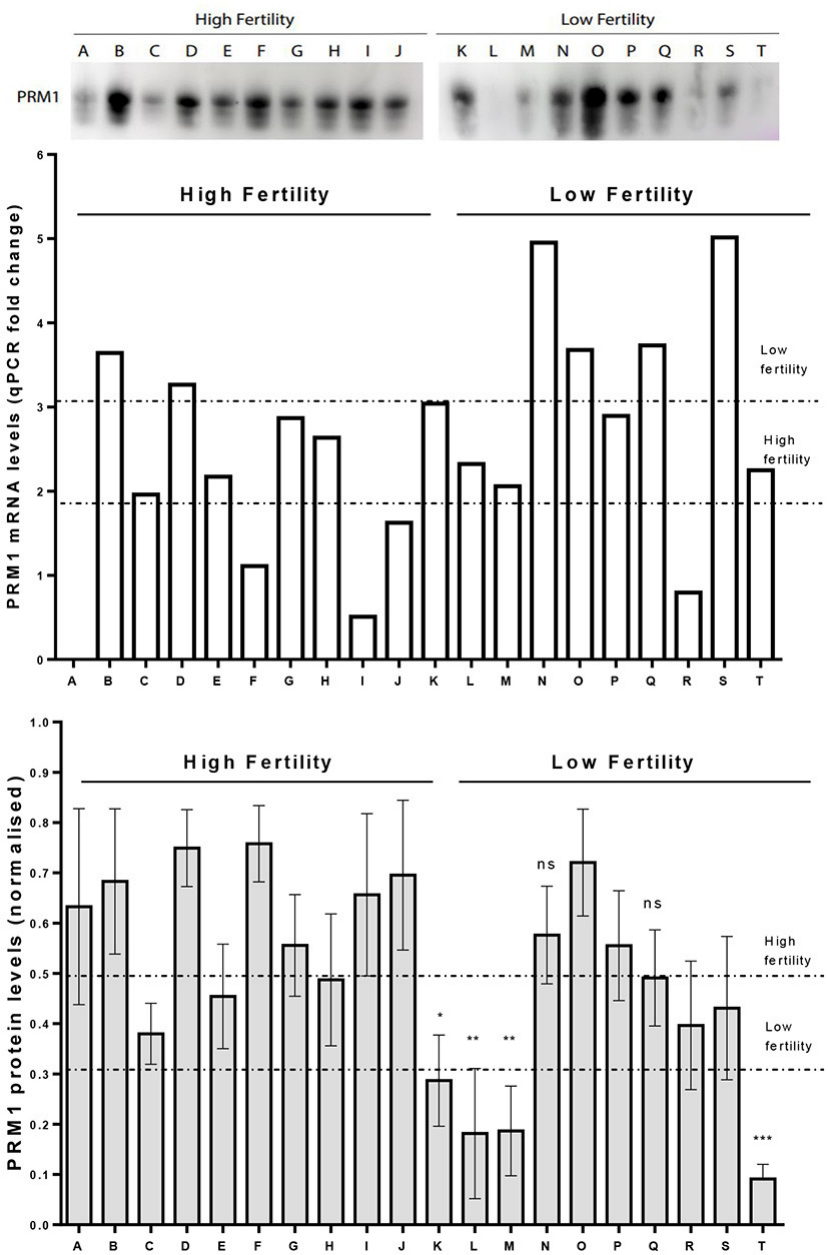
Comparison on PRM1 transcription and protein abundance levels. Upper panel. A representative image for PRM1 protein levels assessed by Western Blot. Mid panel. *PRM1* mRNA levels as revealed by qPCR where bull A was a control comparison. Lower panel. PRM1 protein levels assessed by Western Blot. Low fertility (LF) bulls presented on average, a decrease in the PRM1 protein abundance compared to high fertility (HF) bulls (*p* < 0.05, unpaired *t*-test). The decrease in abundance was particularly evident in 4 LF bulls (K, L, M and T), while the others were similar to HF bulls. * *P* < 0.05; ** *P* < 0.01; *** *P* < 0.001; ns, non-significant.

A western blot analysis was carried out to verify PRM1 protein abundance levels. On average, PRM1 protein levels for the LF group were reduced compared to the HF group (*p* < 0.05; Figure 5), with 9 out of 10 LF bulls having PRM1 protein levels less than the HF average. The reduction in the LF group was mainly driven by four bulls, which presented a significant decrease in PRM1 levels compared to the HF group. PRM1 protein levels were also much more variable in the LF than the HF group.

### Evaluation of protamine deficiency and DNA fragmentation

**Figure 6:**
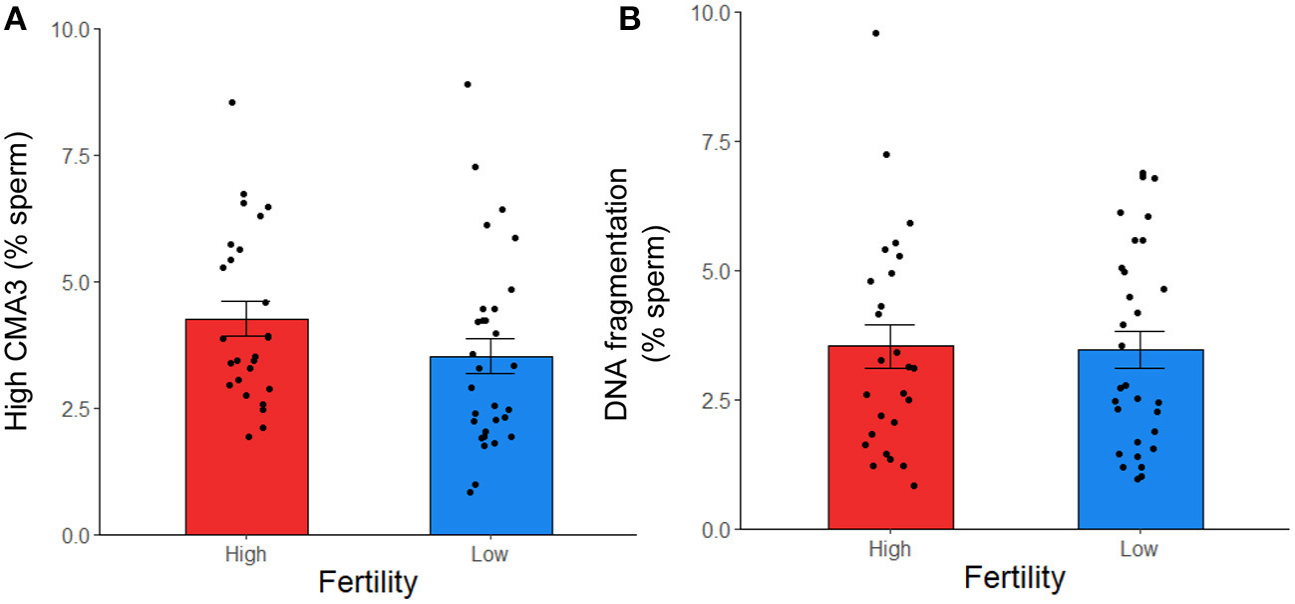
Protamine deficiency (high CMA3) **(A)** and DNA fragmentation **(B)** in frozen-thawed spermatozoa from HF (*n* = 10 bulls; with the exception of high CMA3, where *n* = 9) and LF (*n* = 10) bulls, assessed with CMA3 and AO, respectively. Each data point represents an individual ejaculate from a bull. No differences between HF and LF was observed for these attributes (*p* > 0.05).

**Figure 7:**
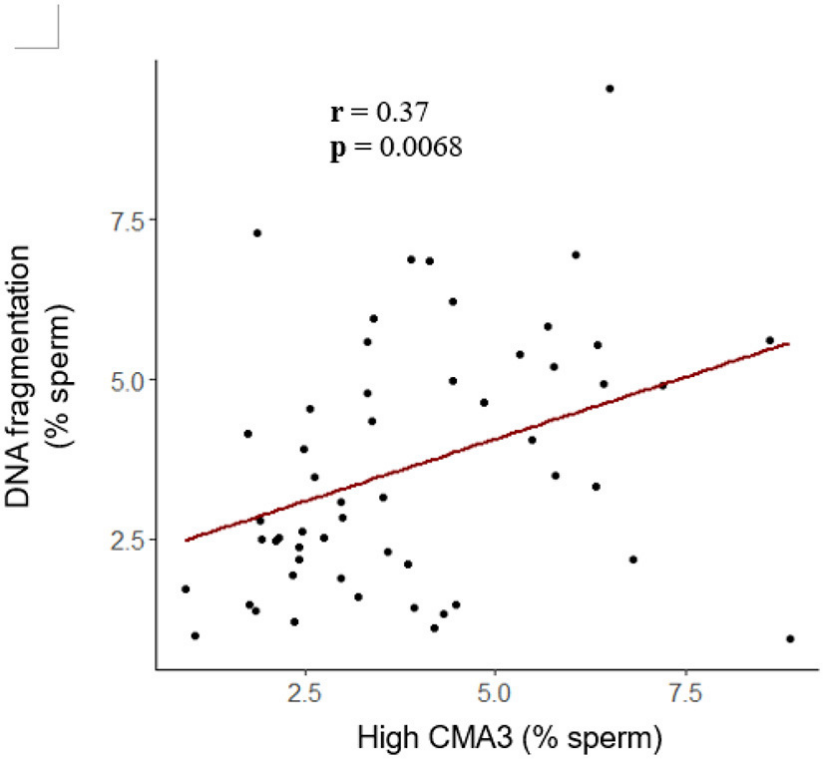
Correlation between the percentage of spermatozoa from bulls with protamine deficiency (high CMA3) and DNA fragmentation. Based on these data, as the percentage of spermatozoa with protamine deficiency increased, so too did the incidence of DNA fragmentation (*r* = 0.37; *p* < 0.01; Pearson's product-moment correlation). Each dot represents an individual ejaculate from a bull of HF (*n* = 9) and LF (*n* = 10).

To further investigate sperm protamine deficiency as well as the incidence of DNA fragmentation, HF and LF bulls were subjected to the flow cytometric assessment of CMA3 and AO, respectively. Overall, there were no differences in the percentage of spermatozoa with protamine deficiency (high CMA3 staining) or DNA fragmentation between HF and LF bulls (*p* > 0.05; Figure 6). A consistent finding across all attributes assessed was the level of variability for both HF and LF (Figure 4). Aside from the absence of differences in these attributes, there was a significant and positive correlation between spermatozoa with protamine deficiency and DNA fragmentation for bulls (and ejaculates), irrespective of fertility group (*p* < 0.01; Figure 7).

## Discussion

The aim of the present study was to identify differentially expressed mRNAs and miRNAs in the spermatozoa of a population of bulls with reliable divergent fertility. Sequencing allowed us to catalog a population of mRNAs present within the bull sperm samples. Among the list of the most highly expressed mRNAs, five already been reported in previous publications: transcripts from genes *PRM1, CHMP5, CCDC18, HMBG4* and *KIF5C* (6, 8, 16). Nevertheless, only six differentially expressed mRNAs were identified in total, which could be explained by several factors. First, the adjusted fertility score used to categorize bulls as HF or LF, is based on an average of 13,292 AIs, carried out across the bull's career, and using multiple different ejaculates. Pooling three different ejaculates for each bull prior to RNA extraction was an attempt to be more representative of an individual bull's average fertility by erasing inter-ejaculate variability in terms of semen quality. Secondly, considering that the low fertility phenotype may have a wide range of underlying causes for each individual bull, combining bulls as two groups is a convenient method for analysis but solely permits the segregation of major differences, and not the most subtle ones. This phenomenon is further strengthened by the relatively high number of bulls in each group for this type of study (*n* = 10 per group) compared to others.

The most highly expressed miRNAs identified in the current study are similar to those described by Sellem, et al. (7), with bta-miR-100 being the most highly expressed miRNA, and 13 out of the 20 miRNAs being common to both studies. This overlap between two studies using completely independent groups of bulls adds validity to the findings. As for the mRNA-seq, the miRNA analysis revealed only 13 differentially expressed miRNAs. Nevertheless, several of the miRNAs are particularly relevant in the context of fertility. In particular, bta-miR-155, had increased expression in the testis of chickens with low sperm motility (52). Similarly, in our study, this miRNA was down-regulated in HF bulls. It is likely to be expressed and brought to the sperm cells by the epithelial cells of the caput epididymis during sperm maturation (53) and is also involved in multiple inflammatory pathologies (54).

Tsatsanis, et al. (55) reported that miR-155 was a potential marker of human subfertility which was in line with its decreased expression in the HF group in this study (55). In comparison, miR-146a, which was also decreased in the HF group, has been described as a beneficial target for improving bull fertility (56), potentially by its action on the low-density lipoprotein receptor (*LDLR*) transcripts. Bta-miR-98, another inflammatory-related miRNA found differentially expressed in our study, was downregulated following infection with bacterial lipopolysaccharide and immune activation in rat testis (57). This miRNA, which plays a role in the inhibition of the anti-apoptotic agent B-cell lymphoma-extra large (*Bcl-xl*) translation, induces an increased cell proliferation during the rat peri-implantation embryo development (58). The miRNA, bta-miR-486, which exhibited increased expression in the HF group, has been described as controlling spermatogonial sperm cells gene expression and growth properties by targeting an activator of the β-catenin signaling pathway. Another miRNA, bta-miR-374b, with decreased expression in the HF group, had a stable expression in the spermatozoa of fertile men (59) but its expression was altered in the seminal plasma of infertile men (60). Interestingly, its expression was markedly decreased in azoospermia, but increased in asthenozoospermia cases.

Notably, three miRNA including bta-miR-655, bta-miR-2285p and bta-miR-2285t were listed as having a target mRNA gene *RBBP6* with increased expression in the HF group. While bta-miR-2285p and bra-miR-2285t have been reported to have roles in the bovine estrous cycle (61, 62), *RBBP6* sperm transcripts were reportedly involved *in utero* embryonic development (63). Analysis of the transcripts targeted by the differentially expressed miRNAs showed that five of them (bta-miR-2285p, bta-miR-98, bta-miR-155, and bta-miR-374b, and bta-miR-486) appear to specifically target elements involved in three major biological pathways. The first one is the signaling pathway regulating pluripotency of stem cells, which encompass the transcription factors and their downstream target gene that promote pluripotent stem cells self-renewal and pluripotency (64). The second one, mammalian target of rapamycin (mTOR) signaling pathway, which exists in two mTOR complexes (mTORC1 and mTORC2), is a central regulator of cell metabolism, growth, proliferation, and survival, and plays a key role during gamete production as well as early embryo development (65). Finally, the FoxO signaling pathway involves a family of transcription factors that regulates the expression of genes implicated in apoptosis, cell-cycle control, glucose metabolism, oxidative stress resistance, and longevity. Knockdown of *FoxO1, FoxO2* and *FoxO4* genes has been shown to impair mouse preimplantation embryonic development (66). In the current study, it is difficult to decipher the collective actions of the differentially expressed miRNAs, but from previous studies it is likely that these miRNAs have an effect on early embryo development, prior to embryonic genome activation (23, 67). Further analyses, carried out on embryos generated with the semen from HF and LF bulls, is needed to corroborate this. Indeed the integration with other molecular component of the cell, such as DNA methylation or histone posttranslational modifications (68) would also be useful.

PRM1 is the most highly expressed mRNA in bovine spermatozoa (6, 8, 69). Terminally replacing the histone DNA binding proteins during spermatogenesis, PRM1 is a core element for the establishment of the highly condensed state of mammalian sperm chromatin. Numerous studies have already shown that perturbation in PRM1 implementation in mature spermatozoa can have deleterious consequences on sperm chromatin structure. For example, in bovine spermatozoa, reduced protamination has been linked to an increase in sperm DNA fragmentation, and a decrease in fertility potential (70). However, strong overexpression of PRM1 protein during spermatogenesis leads to complete sterility in mice due to impaired spermatid maturation, affecting sperm viability and motility (71). In our study, we found that, on average, LF bulls showed significantly reduced PRM1 protein abundance, as revealed by western blotting. This reduced PRM1 protein abundance was associated with a significant increase in *PRM1* mRNA expression, showed by the RNA-seq and RT-qPCR data. Poor quality bull spermatozoa have been shown to have significantly lower levels of *PRM1* mRNAs expression compared that of good quality spermatozoa (11). Similar mRNA results were obtained in human spermatozoa, with *PRM1* mRNA and protein levels found to be positively correlated with sperm concentration, motility, fertilization potential and embryo quality (72, 73). Nevertheless, in other studies, similar results to the current study were obtained, such as an increase in *PRM1* transcripts in low motility human sperm fractions compared to high motile ones (74), or an aberrant *PRM1* transcript retention associated with abnormal protein synthesis in cases of infertile men (75, 76).

One hypothesis to explain this transcript retention phenomenon is that if there is an inefficient translation of the *PRM1* transcripts into proteins during the later stages of spermatogenesis, the transcripts could remain within the spermatozoa, resulting in greater transcript abundance and reduced protein production in the fully mature cell, which would impair fertility. However, the disparity between mRNA and protein abundance of PRM1 could be due to many post-transcriptional modifications (such as microRNA regulation), post-translational modifications (such as methylation, acetylation, etc.), and differential protein degradation (such as proteasome-mediated or autophagy-mediated protein breakdown), the mRNA and protein levels are rarely in line with each other. Moreover, PRM1 content by Western blotting alone is not predictive of bull fertility, because there is not always an adequate amount of PRM1 protein in spermatozoa from LF bulls, but rather an inadequate localization of the protein, in the acrosomal region of the head, which is associated with a distorted nuclear shape (70). More investigation on the nuclear localization of PRM1 proteins, as well as its implementation during the different steps of spermatogenesis, is warranted, but the present result is a good clue for a deeper understanding of bull fertility at the molecular level.

As a means to further investigate the identification of *PRM1* as a biomarker of fertility, we employed the fluorophore CMA3, to indirectly assess protamine deficiency owing to its ability to bind to available G-C sites. These sites are assumed to increase as the level of protamines, and therefore chromatin compaction, decreases (48). Whilst the percentage of cells positive for CMA3 labeling has been used as a predictive tool to evaluate fertility in humans (48, 77) this was not the case for the population of bulls in this study. However, a frequently observed consequence of reduced protamination is the susceptibility of DNA to fragmentation (78), which is primarily associated with an increased production of ROS (78, 79). The incidence of high sperm DNA fragmentation has been linked with reduced embryo quality and implantation in humans (80, 81). Based on our results, protamine deficiency was positively and moderately correlated with the percentage of spermatozoa with DNA fragmentation, irrespective of fertility group.

In conclusion, by assessing mRNAs and miRNAs from the spermatozoa of the same individuals, our results underline the important involvement of PRM1 and miRNAs in the fertility of bulls. Integrating these as biomarkers in the prophylactive screening of bull semen will provide further insight into the underlying biology of unexplained variation in field fertility between bulls demonstrating acceptable spermatozoa functional and morphological characteristics.

## Data availability statement

The datasets presented in this study can be found in online repositories. The names of the repository/repositories and accession number(s) can be found in the article/Supplementary material.

## Ethics statement

Ethical review and approval was not required for the animal study because the semen samples used were collected from bulls under routine commercial practice and later donated to research. Written informed consent was obtained from the owners for the participation of their animals in this study.

## Author contributions

EMDo identified the bulls, procured the bull semen and drafted the manuscript with J-PP. J-PP performed the extraction of total RNAs, the quality controls before sequencing and the validations by qPCR. KK performed the bioinformatics and statistical analyses of mRNA and miRNA-seq data. MŠ and NCB completed the flow cytometry analysis. CMC and EMDu performed the western blot assay. DAK, SF, and PL conceived and obtained funding for the study and performed supervision of the work as well as critical revision of the manuscript. ES provided the RNA extraction protocol, performed quality controls by qPCR, and gave advice for the project realization. All authors have read and approved the final manuscript.

## Funding

This research was funded by Science Foundation Ireland under the Investigators Programme (16/IA/4474). EMDo was funded by the Irish Research Council (Project GOIPG/2017/1884). CMC was funded by a Post-Doctoral Fellowship from the National University of Ireland (NUI). EMDu was funded by Science Foundation Ireland-PIYRA 13/YI/2187.

## Conflict of interest

Author ES was employed by ALLICE. The remaining authors declare that the research was conducted in the absence of any commercial or financial relationships that could be construed as a potential conflict of interest.

## Publisher's note

All claims expressed in this article are solely those of the authors and do not necessarily represent those of their affiliated organizations, or those of the publisher, the editors and the reviewers. Any product that may be evaluated in this article, or claim that may be made by its manufacturer, is not guaranteed or endorsed by the publisher.
