# Supplementary material for: Identification of differentially expressed mRNAs and miRNAs in spermatozoa of bulls of varying fertility
Source: Front Vet Sci. 2022 Oct 5;9:993561. doi: 10.3389/fvets.2022.993561 (PMC9581129; doi:10.3389/fvets.2022.993561)
Supplement: Supplementary file 1 [file Data_Sheet_1.docx]

To review the mRNA data

Go to <https://scanner.topsec.com/?d=1452&r=show&u=https%3A%2F%2Fwww.ncbi.nlm.nih.gov%2Fgeo%2Fquery%2Facc.cgi%3Facc%3DGSE198043&t=b9452d09f4bdd8ed9672204296746d8776150b68>

Enter the GEO accession GSE198043

Enter token udktckqstrwjhal into the secure token box

To review the miRNA data

Go to <https://scanner.topsec.com/?d=1452&r=show&u=https%3A%2F%2Fwww.ncbi.nlm.nih.gov%2Fgeo%2Fquery%2Facc.cgi%3Facc%3DGSE198043&t=b9452d09f4bdd8ed9672204296746d8776150b68>

Enter the GEO accession GSE196750

Enter token gterewsqdhqjhwh into the secure token box
