# Supplementary material for: Identification of differentially expressed mRNAs and miRNAs in spermatozoa of bulls of varying fertility
Source: Front Vet Sci. 2022 Oct 5;9:993561. doi: 10.3389/fvets.2022.993561 (PMC9581129; doi:10.3389/fvets.2022.993561)
Supplement: Supplementary file 2 [file Data_Sheet_2.docx]

| **Table S1.** Population of bulls used for the study. | | |  |
| --- | --- | --- | --- |
| AI code | Number of inseminations | Adjusted fertility | Fertility category |
| 1 | 8637 | 0.060 | High |
| 2 | 37849 | 0.067 | High |
| 3 | 11459 | 0.058 | High |
| 4 | 3912 | 0.065 | High |
| 5 | 12424 | 0.068 | High |
| 6 | 100288 | 0.068 | High |
| 7 | 17441 | 0.062 | High |
| 8 | 5119 | 0.062 | High |
| 9 | 34973 | 0.071 | High |
| 10 | 1041 | 0.067 | High |
| 11 | 23811 | -0.043 | Low |
| 12 | 740 | -0.091 | Low |
| 13 | 519 | -0.093 | Low |
| 14 | 1034 | -0.085 | Low |
| 15 | 1477 | -0.123 | Low |
| 16 | 1772 | -0.073 | Low |
| 17 | 1195 | -0.035 | Low |
| 18 | 568 | -0.030 | Low |
| 19 | 597 | -0.046 | Low |
| 20 | 980 | -0.039 | Low |

| **Table S2.** RT-PCR primers for quality controls after RNA extraction. | | |  |  |  |
| --- | --- | --- | --- | --- | --- |
| Primer name | Gene symbol  (Ensembl ID) | Accession number | Primer sequence (5'-3') | Primer length  (bp) | Product size  (bp) |
| PRM1_F | PRM1 (ENSBTAG00000021493) | NM_174156.2 | AAGATGTCGCAGACGAAGGAG | 21.00 | 222.00 |
| PRM1_R |  |  | GTGGCATTGTTCGTTAGCAGG | 21.00 |  |
| PTPRC_F | PTPRC (ENSBTAG00000023144) | NM_001206523.1 | ACCCAACCTTCTACTCAAGATG | 22.00 | 124.00 |
| PTPRC_R |  |  | CGTATTTGTTCTCACATGGTGG | 22.00 |  |
| CDH1_F | CDH1 (ENSBTAG00000015991) | NM_001002763.1 | CTGCATTCCTGGCTTTGGTG | 20.00 | 171.00 |
| CDH1_R |  |  | GTAAGCACGCCATCTGTGTG | 20.00 |  |
| KIT_F | KIT (ENSBTAG00000002699) | NM_001166484.1 | GAATAGCTGGCATCAGGGTG | 20 | 224 |
| KIT_R |  |  | CCAGATCCACATTCTCTCCATC | 22 |  |
| From Selvaraju et al., 2017 | |  |  |  |  |

| **Table S3.** RT-qPCR primers for validations of the mRNA-seq data. | | |  |  |  |
| --- | --- | --- | --- | --- | --- |
| Primer name | Gene symbol  (Ensembl ID) | Accession number | Primer sequence (5'-3') | Primer length  (bp) | Product size  (bp) |
| PRM1_F1 | PRM1 (ENSBTAG00000021493) | NM_174156.2 | AAGATGTCGCAGACGAAGGAG | 21.00 | 222.00 |
| PRM1_R1 |  |  | GTGGCATTGTTCGTTAGCAGG | 21.00 |  |
| SCP2D1_F1 | SCP2D1 (ENSBTAG00000005202) | NM_001040507.2 | GGCAAGTTCAAAGTGAGCGG | 20.00 | 98.00 |
| SCP2D1_R1 |  |  | TTCCTGATATTCCGGCAGGC | 20.00 |  |
| SLC14A1_F1 | SLC14A1 (ENSBTAG00000025826) | NM_174655.2 | CACGAGCAGACCCACTCTT | 19.00 | 98.00 |
| SLC14A1_R1 |  |  | AGAGGGTAACCACAAACCTTCA | 22.00 |  |
| SLC14A1_F2 | SLC14A1 (ENSBTAG00000025826) | NM_174655.2 | CACGAGCAGACCCACTCTTTG | 21.00 | 88.00 |
| SLC14A1_R2 |  |  | CACAAACCTTCAAACCTGGACAT | 23.00 |  |
| RBBP6_F1 | RBBP6 (ENSBTAG00000009441) | NM_001304554.1 | ACAAGCACCACCTTTGTCCA | 20.00 | 77.00 |
| RBBP6_R1 |  |  | TGGACTTTTTCTTTTCCTCTTCTTT | 25.00 |  |
| CHD9_F1 | CHD9 (ENSBTAG00000002287) | NM_001205650.2 | CCTCTAGGGCGCAGACGAAA | 20.00 | 114.00 |
| CHD9_R1 |  |  | ATGAAGTGTCAGAATCCTTGGCT | 23.00 |  |
| NovelGene1_F1 | Novel Gene 1 (ENSBTAG00000048468) | / | ACCCTTCTCCCAACCAGTGA | 20.00 | 164.00 |
| NovelGene1_R1 |  |  | CTGAAGCTTGGCCCTTCTTG | 20.00 |  |
| NovelGene1_F2 | Novel Gene 1 (ENSBTAG00000048468) | / | CGTTGCCTTAACAGGTGGTG | 20.00 | 129.00 |
| NovelGene1_R2 |  |  | GAAGCTTGGCCCTTCTTGTTC | 21.00 |  |
| NovelGene2_F1 | Novel Gene 2 (ENSBTAG00000054826) | / | TAGGGTGATGGTGGCCTCATA | 21.00 | 199.00 |
| NovelGene2_R1 |  |  | CAGACCCATCACAAGCATGG | 20.00 |  |
| NovelGene2_F2 | Novel Gene 2 (ENSBTAG00000054826) | / | TCAGCTGTGAAGCCGTCTG | 19 | 86 |
| NovelGene2_R2 |  |  | AGACCCATCACAAGCATGGAA | 21 |  |
